# Comparison of Alkane Distributions in Crude Oils Detected by Gas Chromatography of Whole Oils and Saturated Fractions and Gas Chromatography–Mass Spectrometry of Saturated Fractions

**DOI:** 10.1021/acsomega.6c06134

**Published:** 2026-10-19

**Authors:** Wenqiang Wang, Xiaolin Lu, Jiahui Xi, Xingying Wang, Yang You, Xuening Qi, Kun Tian, Peng Song, Xinyu Ai, Chenghan Li, Zhaohua Xue, Baohong Shi

**Affiliations:** † School of Earth Sciences and Engineering, Xi’an Petroleum University, Xi’an 710065, China; ‡ Shaanxi Key Laboratory of Petroleum Accumulation Geology, Xi’an Petroleum University, Xi’an 710065, China; § 124175 SINOPEC Petroleum Exploration and Production Research Institute, Beijing 100083, China; ∥ No.12 Oil Production Plant, Changqing Oilfield Company, Qingyang 745400, China; ⊥ 118425 Exploration and Development Research Institute of PetroChina Changqing Oilfield Company, Xi’an 710018, China; # Natural Gas Research Institute of Yanchang Petroleum (Group) Co.Ltd., Xi’an 710075, China; ∇ Research Institute of Petroleum Exploration and Development, 205361 PetroChina, Beijing 100083, China

## Abstract

Alkane-related parameters of oils obtained by both gas chromatography (GC) and gas chromatography–mass spectrometry (GC-MS) were widely used in oil-source correlation. However, it is still unclear whether different detection methods will lead to significant differences in these parameters. The compositions of alkanes in Jurassic oils from the Sichuan Basin were investigated using GC analysis of whole-oil samples and saturated hydrocarbons and GC-MS analysis of saturated hydrocarbons. Comparative analyses of alkane-related parameters obtained by these detection methods were also performed in this study. *n*-Alkanes in the range *n*C_3_–*n*C_35_ were detected in oils using whole-oil GC analysis. By contrast, short-chain *n*-alkanes (*n*C_3_–*n*C_13_) were not detected by GC and GC-MS analysis of saturated hydrocarbons. The abundances of *n*-alkanes in the range *n*C_14_–*n*C_16_ were lower than those detected by whole-oil GC analysis. *n*C_21_^–^/*n*C_22_^+^ ratios and terrigenous/aquatic ratios (TAR = (*n*C_27_ + *n*C_29_ + *n*C_31_)*/*(*n*C_15_ + *n*C_17_ + *n*C_19_)) obtained by GC analysis of saturated hydrocarbons are similar to those obtained by *m*/*z* 85 mass chromatograms of GC-MS and show huge differences from those obtained by whole-oil GC analysis and total ion current of GC-MS. However, the differences between these ratios obtained by GC analysis of saturated hydrocarbons, *m*/*z* 85 mass chromatograms, and total ion current of GC-MS are systematic. Pristane/phytane (Pr/Ph) ratios obtained by GC analysis of saturated hydrocarbons exhibit little difference from those obtained by total ion current of GC-MS but show large differences from those obtained by whole-oil GC and *m*/*z* 85 mass chromatograms of GC-MS. While, the differences between Pr/Ph obtained by all these methods mentioned above are systematic and can be basically eliminated. Additionally, the different detection methods have no influence on carbon preference index, odd-to-even preference, Pr/*n*C_17_ and Ph/*n*C_18_.

## Graphical abstract

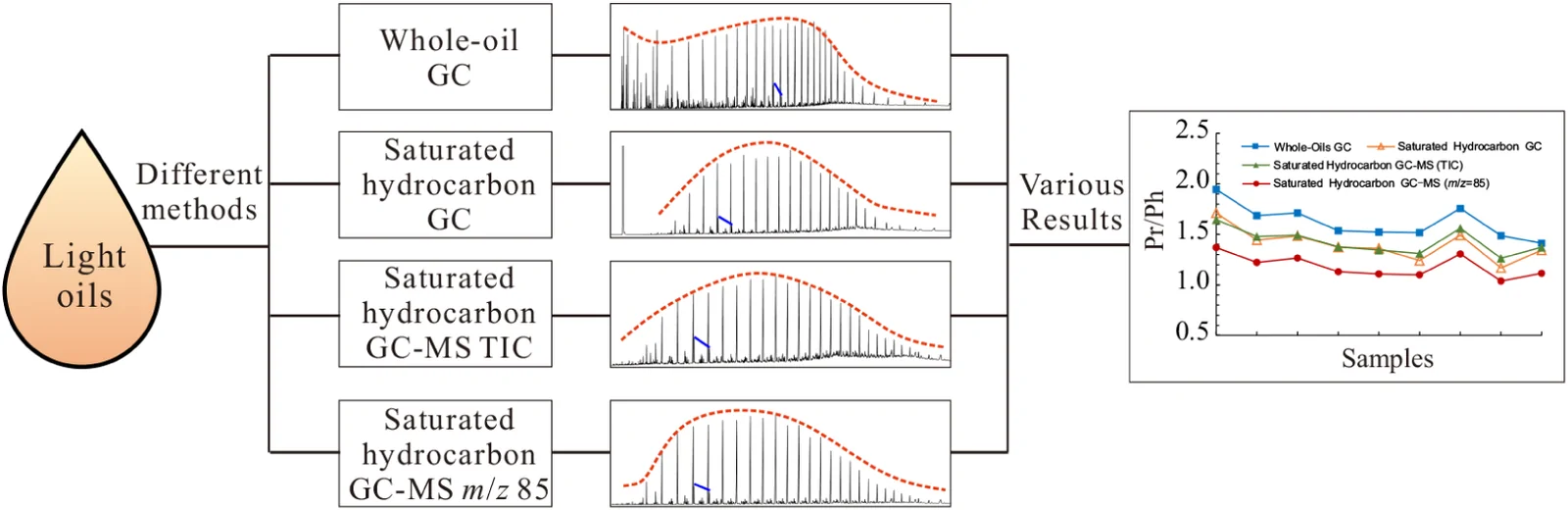

## 1 Introduction

Alkanes are abundant in crude oils, including *n*-alkanes and acyclic isoprenoid alkanes.^1,2^ The distribution patterns of *n*-alkanes in crude oils can provide geochemical information on organic matter sources, thermal maturity, and secondary alteration processes.^3^ Oils derived predominantly from higher plants typically exhibit relatively higher abundances of long-chain *n*-alkanes, with a unimodal, back-end dominated distribution. In contrast, a unimodal, front-end dominated *n*-alkane distribution generally indicates that the organic matter source is dominated by lower aquatic organisms such as algae.^4^ Low-maturity oils derived from higher plants commonly show pronounced odd- or even-carbon number predominance in their *n*-alkane distributions.^5^ With increasing maturity, carbon–carbon bonds break, leading to an increase in low-molecular-weight *n*-alkanes and the disappearance of the odd/even predominance.^6^ Furthermore, secondary alterations such as biodegradation can significantly affect the abundances and distribution patterns of *n*-alkanes; thus, *n*-alkane distributions are also widely used to assess the degree of biodegradation.^7,8^ Pristane and phytane are considered to be mainly derived from the chlorophyll of photosynthetic organisms, and their ratio (Pr/Ph) is a common parameter for indicating depositional environments.^1,9^

Whole-oil gas chromatography (whole-oil GC), saturated hydrocarbon GC, and saturated hydrocarbon GC-mass spectrometry (GC-MS) are all routinely used to detect and identify alkanes in crude oils.^10^ Whole-oil GC analyzes the unseparated crude oil. For crude oils*—*especially light oils and condensates*—*this method not only reveals the distribution patterns of long-chain alkanes but also preserves the information on light hydrocarbon components (C_4_–C_13_ low-molecular-weight hydrocarbons) relatively completely.^11,12^ To increase the concentrations of biomarker compounds for analytical convenience, crude oils are often fractionated into saturated hydrocarbons, aromatic hydrocarbons, resins, and asphaltenes.^13^ However, because light hydrocarbons are easily volatilized,^14^ a significant portion of them is lost during fractionation. Consequently, compared to whole-oil GC, saturated hydrocarbon GC inevitably yields lower abundances of low-carbon-number *n*-alkanes. The total ion current (TIC) chromatogram and the *m*/*z* 85 mass chromatogram from saturated hydrocarbon GC-MS are also commonly used to identify *n*-alkanes and acyclic isoprenoid alkanes.^15–17^

It is often assumed that the distribution patterns observed in saturated hydrocarbon GC, the TIC of saturated hydrocarbon GC-MS, and the *m*/*z* 85 mass chromatogram are broadly similar. However, increasing evidence indicates that geochemical parameters derived from different analytical platforms are not necessarily directly comparable. Wang et al. (2011)^18^ compared 23 petroleum geochemical parameters obtained by GC×GC-TOFMS and conventional GC-MS and found that only 10 parameters showed deviations of less than 5%, whereas substantial discrepancies occurred for the other 13 parameters. French et al. (2020)^19^ further demonstrated that the Carbon Preference Index (CPI) showed good comparability between GC-QQQ-MS and previously reported GC-FID measurements, whereas the comparability of ratios involving Pr, Ph, and/or *n*-alkanes was more variable. These observations indicate that a geochemical parameter obtained by one analytical method cannot automatically be regarded as equivalent to the same nominal parameter obtained by another method.

This issue is particularly relevant to light oils and condensates, because low-molecular-weight hydrocarbons carry important geochemical information but are also susceptible to evaporative loss during sample handling and group-type fractionation.^11–14^ In practical petroleum geochemical studies, alkane data may be generated in different analytical batches or obtained using different analytical workflows. However, although previous studies have demonstrated that analytical procedures and instrumental platforms can influence measured geochemical parameters, the parameter-specific comparability among whole-oil GC, saturated hydrocarbon GC, and different GC-MS chromatographic outputs has not been systematically evaluated. This is particularly important because alkane-derived parameters, such as *n*C_21_^–^/*n*C_22_^+^, the CPI,^20^ the OEP^21^ and the Pr/Ph ratio, are widely used in petroleum geochemical interpretation.^22,23^ Consequently, an important practical question arises: when different analytical workflows yield different values for the same geochemical parameter, which results can be reliably compared or used interchangeably without altering the geological interpretation? Meanwhile, routine oilfield studies also require efficient and streamlined analytical workflows. It is therefore practically important to determine which alkane-related parameters can be reliably obtained using GC measurements alone, and which parameters require GC-MS analysis, method-specific treatment, or calibration.

In this study, nine crude oil samples from the Sichuan Basin were analyzed by whole-oil GC, saturated hydrocarbon GC, and saturated hydrocarbon GC-MS. Rather than simply comparing alkane distribution patterns obtained by different analytical methods, this study focuses on the practical comparability and applicability of commonly used alkane-related geochemical parameters. Specifically, we evaluate (1) which parameters are strongly method-dependent and therefore should not be directly mixed among data sets generated by different analytical workflows; (2) which parameters show sufficient consistency between GC and GC-MS measurements and can therefore be used interchangeably; and (3) for which parameters a GC-based workflow can provide sufficiently reliable information for routine petroleum geochemical applications without requiring additional GC-MS analysis. By establishing parameter-specific criteria for data comparability, this study provides a practical basis for integrating heterogeneous geochemical data sets and for selecting appropriate analytical workflows in oil-source and oil–oil correlation studies.

## 2 Samples and Experimental Methods

### 2.1 Sample Information

Nine crude oil samples from the Jurassic system in the Sichuan Basin were collected for this study. Among these, two samples are from the Da’anzhai Member of the Ziliujing Formation in the Gongshanmiao oilfield, two from the Lianggaoshan Formation, three from the first member of the Shaximiao Formation, one from the Da’anzhai Member in the Jinhua oilfield, and one from the Da’anzhai Member in the Bajiaochang area (Table 1). The crude oil samples are predominantly light oils, with densities ranging from 0.8270 to 0.8495 g/cm^3^. In terms of bulk composition, saturated hydrocarbons are the most abundant fraction, followed by aromatic hydrocarbons, with asphaltenes being the least abundant. Specifically, saturated hydrocarbons account for 75.47%–85.93% (average 79.95%), while aromatic hydrocarbons range from 8.64% to 18.59% (average 13.24%). Light-hydrocarbon and carbon-isotopic compositional data reveal that crude oils from the Da’anzhai Member, Lianggaoshan Formation, and the first member of the Shaximiao Formation within the study area are mature oils belonging to one oil family, and are all sourced from the Da’anzhai Member and Lianggaoshan Formation source rocks.^24,25^

**Table 1:** Gross Composition of Jurassic Oils from the Sichuan Basin

No.	Well	Depth (m)	Formation	Sat. (%)	Aro. (%)	Nonhydro. (%)	Asph. (%)	Sat/Aro
1	G16	2131.0–2154.0	Shaximiao	75.47	16.12	3.97	4.44	4.68
2	GH16	2138.8–2145.7	Shaximiao	85.93	8.64	3.95	1.48	9.94
3	GH18	2106.9–2110.1	Shaximiao	83.18	11.36	4.77	0.68	7.32
4	G101	2615.0–2688.6	Lianggaoshan	81.70	13.14	4.33	0.83	6.22
5	G13	2401.6–2427.2	Lianggaoshan	79.19	14.09	5.37	1.34	5.62
6	G108x	2536.5–2540.6	Da’anzhai	79.08	12.41	5.52	2.99	6.37
7	G111	2561.7–2594.4	Da’anzhai	76.69	11.65	5.42	6.23	6.58
8	JN35	2522.6–2525.0	Da’anzhai	76.44	18.59	3.14	1.83	4.11
9	J94	2540.4–2609.0	Da’anzhai	81.84	13.10	3.22	1.84	6.25

### 2.2 Experimental Conditions

All crude oil samples were collected at wellhead separators and were immediately sealed and refrigerated upon collection. To minimize the loss of light hydrocarbon components, whole-oil gas chromatography (whole-oil GC) was performed promptly after sample collection. Whole-oil GC was conducted using an Agilent 6890 gas chromatograph, with analytical procedures implemented in accordance with the Chinese industry standard Analytical method of hydrocarbons in petroleum and sediment by gas chromatography (SY/T 5779–2024). Helium was used as the carrier gas at a flow rate of 1.0 mL/min. An HP-PONA fused silica capillary column (50 m × 0.2 mm × 0.5 μm) was employed. The temperature program was as follows: initial temperature 35 °C, hold for 5 min; ramp to 70 °C at 3 °C/min; then ramp to 300 °C at 4.5 °C/min, and hold for 35 min. The FID detector temperature was set to 300 °C, with hydrogen and air flow rates controlled at 40 mL/min and 400 mL/min, respectively.

For group-type fractionation of crude oils, approximately 30 mg of each sample was first dissolved in excess petroleum ether to remove asphaltenes. Subsequently, saturated hydrocarbons, aromatic hydrocarbons, and resins were eluted sequentially from a silica gel/alumina (3:2, v/v) chromatographic column using petroleum ether, a mixture of petroleum ether and dichloromethane (2:1, v/v), and a mixture of dichloromethane and methanol (93:7, v/v), respectively. Saturated hydrocarbon GC analysis was performed on a Shimadzu GC-2010 gas chromatograph equipped with a fused silica capillary column (30 m × 0.25 mm × 0.25 μm). Helium was used as the carrier gas at a flow rate of 1.0 mL/min. The injector temperature was 300 °C. The column oven was initially set to 100 °C, held for 1 min, then ramped to 300 °C at 4 °C/min and held for 25 min. The FID detector temperature was 300 °C, and the hydrogen and air flow rates were the same as those used in whole-oil GC analysis. Saturated hydrocarbon GC analysis was performed in accordance with the Chinese national standard *Organic geochemical analysis methods for geological samples—Part 5: Analysis of saturated hydrocarbons in rock extracts and crude oils—Gas chromatography* (GB/T 18340.5–2010).

Saturated hydrocarbon gas chromatography–mass spectrometry (GC-MS) was conducted on an Agilent 6890 GC/5975i MSD instrument, with analytical procedures following the Chinese national standard *The test method for biomarkers in sediment and crude oil by GC-MS* (GB/T 18606–2017). The helium carrier gas flow rate was 1 mL/min. An HP-5MS fused silica capillary column (30 m × 0.25 mm × 0.25 μm) was used. The injector temperature was maintained at 300 °C. The GC temperature program was: initial temperature 50 °C, hold 1 min; ramp to 120 °C at 20 °C/min; then ramp to 310 °C at 3 °C/min, and hold isothermal for 25 min. The mass spectrometer was operated in electron impact (EI) ionization mode at 70 eV. Data acquisition was performed in both full scan (SCAN) and selected ion monitoring (SIM) modes. All experimental procedures described above were carried out at the State Key Laboratory of Petroleum Resources and Engineering, China University of Petroleum (Beijing).

It should be noted that whole-oil GC, saturated hydrocarbon GC, and saturated hydrocarbon GC-MS represent established analytical workflows for different petroleum-geochemical purposes. Therefore, the differences in capillary columns, temperature programs, and detector systems among these methods reflect the analytical requirements of the respective workflows rather than arbitrary experimental settings. The present study compares the geochemical parameters generated by these routinely applied analytical workflows rather than isolating the effect of a single chromatographic variable under identical instrumental conditions.

## 3 Results and Discussion

### 3.1 n-Alkane Distributions

Whole-oil GC detected 32 *n*-alkanes ranging from *n*C_3_ to *n*C_35_, with very abundant low-molecular-weight *n*-alkanes in the *n*C_3_–*n*C_13_ range. The light hydrocarbon compounds from *n*C_4_ to *n*C_7_, which are widely used in oil correlation and source studies,^26^ show distinct peaks prior to a retention time of 12.5 min (Figure 1a). Saturated hydrocarbon GC detected 22 *n*-alkanes from *n*C_14_ to *n*C_35_, but essentially no *n*-alkanes in the *n*C_3_–*n*C_13_ range. For *n*C_14_–*n*C_16_ *n*-alkanes, their abundances decrease markedly with decreasing carbon number, a trend not observed in the whole-oil GC chromatogram (Figure 1b). On the total ion current (TIC) chromatogram and the *m*/*z* 85 mass chromatogram from saturated hydrocarbon GC-MS, the *n*-alkane distributions are generally similar to those on the saturated hydrocarbon GC chromatogram (Figure 1c, d); the only difference is that pristane and phytane show relatively lower abundances on the *m*/*z* 85 mass chromatogram.

**Figure 1:**
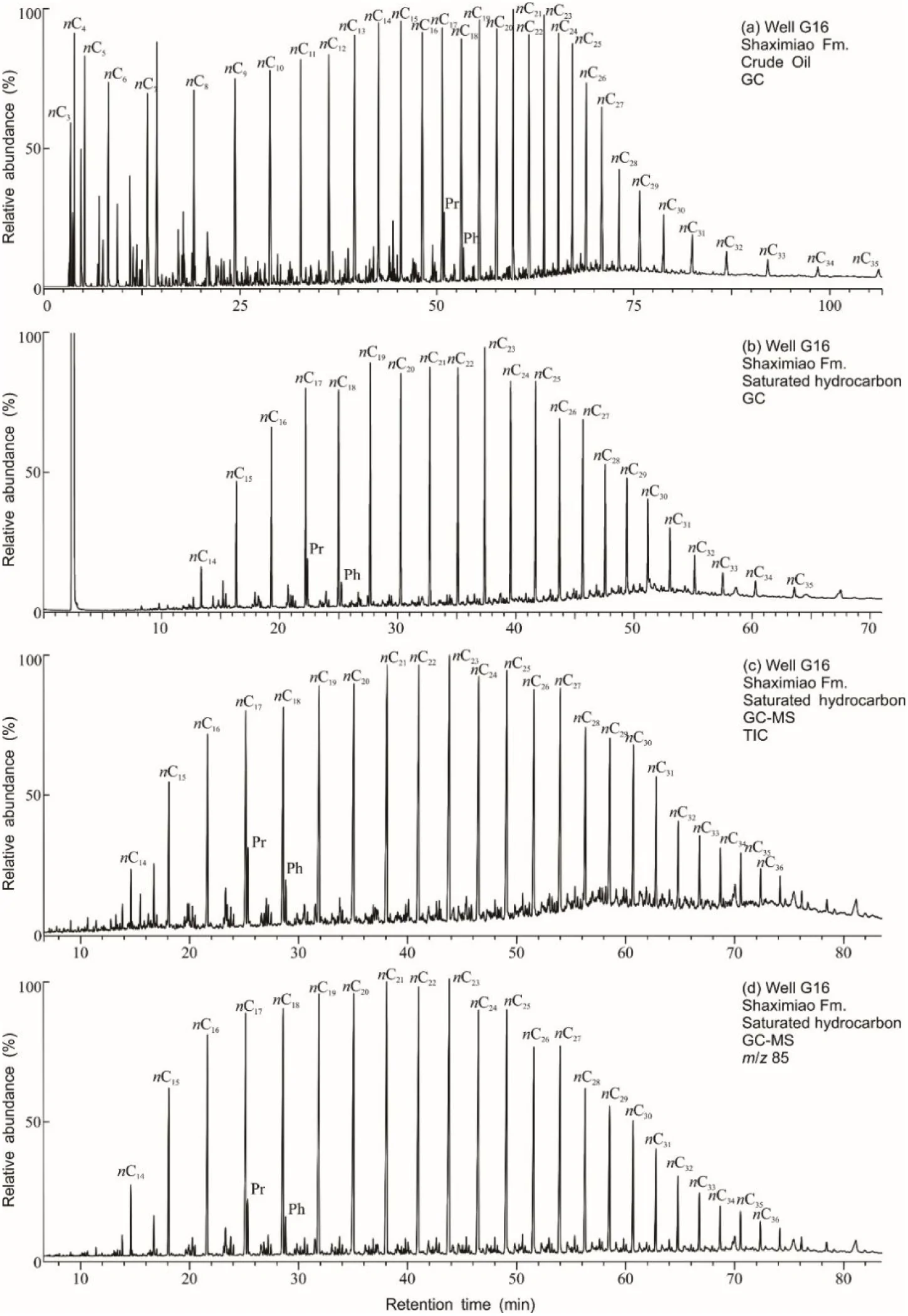
Distribution of *n*-alkanes in Jurassic oils from the Sichuan Basin.

The relative abundance distributions of *n*-alkanes for all samples indicate that the crude oils in the study area exhibit front-dominated *n*-alkane patterns. The predominant peak carbon number for most samples is *n*C_7_, with some samples showing *n*C_15_ or *n*C_23_ as the main peak (Figure 2a). The *n*-alkane distribution patterns displayed on saturated hydrocarbon GC chromatograms, TIC chromatograms, and *m*/*z* 85 mass chromatograms are similar to each other (Figure 2b, c, d). Compared to whole-oil GC, the saturated hydrocarbon-based chromatograms lack *n*C_3_–*n*C_12_, are partially missing *n*C_13_–*n*C_17_, and show decreasing loss with increasing carbon number, with no significant loss observed for *n*-alkanes above *n*C_16_ or *n*C_17_. The pronounced depletion of low-carbon-number *n*-alkanes in the saturated-hydrocarbon-based analyses is consistent with the preferential loss of volatile hydrocarbons during group-type fractionation. Low-molecular-weight hydrocarbons have low boiling points and are easily volatilized during asphaltene precipitation and elution of the saturated hydrocarbon fraction with petroleum ether through the chromatographic column. To determine the carbon number range affected by fractionation, we systematically compared two sets of ratios, *n*C_14_/*n*C_13_, *n*C_15_/*n*C_14_, *n*C_16_/*n*C_15_, *n*C_17_/*n*C_16_, and *n*C_18_/*n*C_17_, obtained from whole-oil GC versus saturated hydrocarbon GC. The ratios derived from whole-oil GC are all approximately 1.0. In contrast, the *n*C_14_/*n*C_13_ ratios calculated from saturated hydrocarbon GC are much larger, ranging from 1.75 to 19.19. These ratios show a clear decreasing trend from low to high carbon numbers, generally approaching 1.0 at *n*C_17_/*n*C_16_ (Figure 3). This indicates that group-type fractionation significantly affects the relative abundances of *n*-alkanes with carbon numbers below 16, but has little effect on the relative abundances of higher molecular weight *n*-alkanes. However, because whole-oil GC and saturated-hydrocarbon-based analyses also differ in chromatographic and instrumental conditions, the respective contributions of sample pretreatment and analytical conditions to the observed discrepancies cannot be quantitatively separated using the present experimental design. Therefore, the observed differences are interpreted here as differences among complete analytical workflows rather than as effects caused exclusively by group-type fractionation.

**Figure 2:**
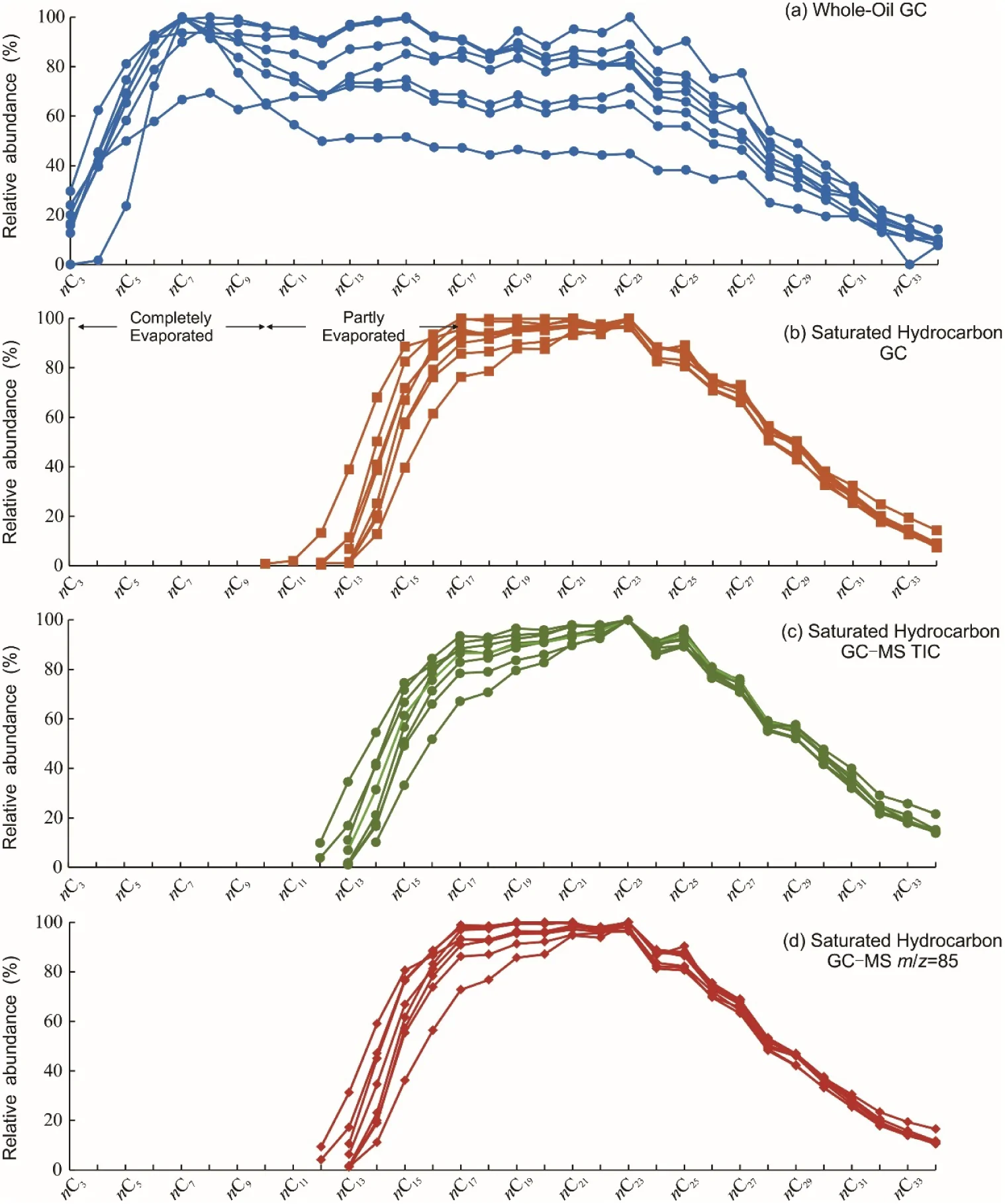
Line charts of relative abundance of C_3_–C_34_ *n*-alkanes in Jurassic oils from the Sichuan Basin.

**Figure 3:**
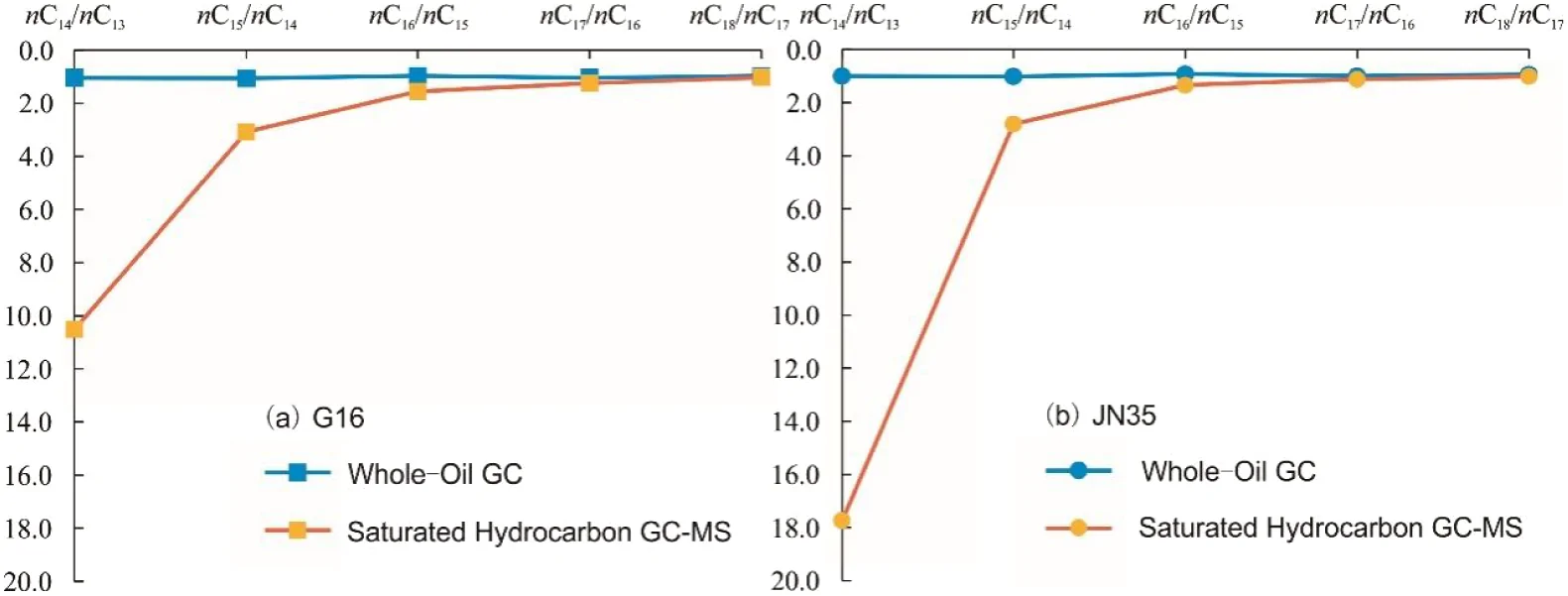
Line charts show the variation of five ratios obtained by GC analysis of whole oils and saturated hydrocarbons in Jurassic oils from wells G16 (a) and JN35 (b) of the Sichuan Basin.

Because group-type fractionation substantially affects the abundances of low-carbon-number *n*-alkanes, it markedly alters the *n*-alkane distribution pattern. For example, whole-oil GC shows that most oil samples have a main peak at *n*C_7_, whereas on saturated hydrocarbon distribution plots the main peak shifts to *n*C_17_–*n*C_23_. Such differences in *n*-alkane distribution patterns inevitably lead to large discrepancies in *n*-alkane-derived parameters, such as the *n*C_21_^–^/*n*C_22_^+^ ratio. To evaluate the influence of different analytical methods (whole-oil GC, saturated hydrocarbon GC, and GC-MS) on commonly used alkane-related parameters, we systematically compared the TAR, *n*C_21_^–^/*n*C_22_^+^, Pr/Ph, CPI, OEP, Pr/*n*C_17_, and Ph/*n*C_18_ obtained from these different methods.

### 3.2 TAR and *n*C_21_^–^/*n*C_22_^+^ Ratios

Certain *n*-alkane ratios can be used to assess the relative abundances of terrestrial versus aquatic organic matter in source rocks and related crude oils. A high Terrestrial/Aquatic Ratio (TAR) indicates a predominantly terrestrial source input, where TAR = (*n*C_27_ + *n*C_29_ + *n*C_31_)/(*n*C_15_ + *n*C_17_ + *n*C_19_).^5,27^ As shown in Figure 4a, the variation trends of TAR values obtained by different analytical methods are generally consistent across samples. TAR values calculated from whole-oil GC are the lowest overall, which is likely because the whole oil samples were not subjected to group-type fractionation, and thus the denominator components (*n*C_15_, *n*C_17_, *n*C_19_) were not lost, resulting in lower TAR values compared to those from other methods. TAR values calculated from the total ion current (TIC) chromatogram of saturated hydrocarbon GC-MS are the highest, while those derived from saturated hydrocarbon GC and from the *m*/*z* 85 mass chromatogram are comparable to each other. If the TAR values obtained from whole-oil GC are taken as the reference standard, then the TAR values calculated from the TIC chromatogram of saturated hydrocarbon GC-MS show deviations ranging from 22.9% to 57.5%, with an average deviation of 41.9% (Table 2).

**Figure 4:**
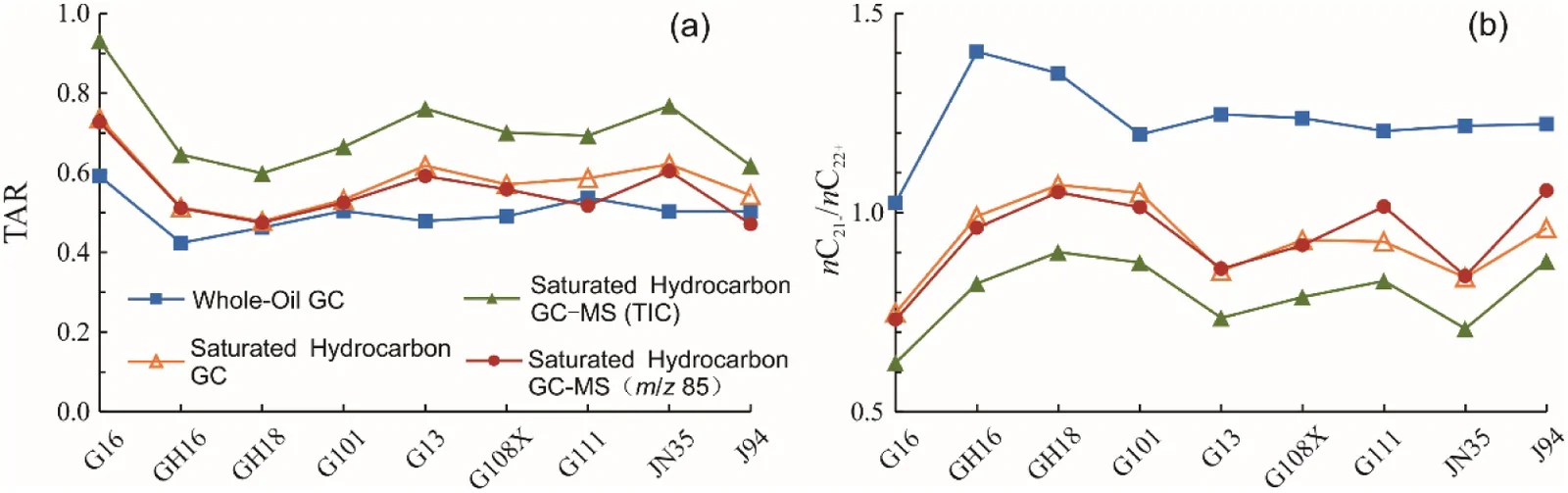
Line charts show the variation of TARs (a) and *n*C_21_^–^/*n*C_22_^+^ ratios (b) in Jurassic oils from the Sichuan Basin.

**Table 2:** Selected Alkane Related Parameters Obtained by Different Detection Methods in Jurassic Oils from the Sichuan Basin^a^

Well	Formation	Sample	Methods	TAR	Relative Deviation (%)	TAR′	Relative Deviation (%)	*n*C_21_^–^/*n*C_22_^+^	Relative Deviation (%)	Pr/Ph	Relative Deviation (%)	CPI	OEP	Pr/*n*C_17_	Ph/*n*C_18_
G16	Shaximiao	Whole-oil	GC	0.59	0.00	/	/	1.02	0.00	1.95	0.00	1.13	1.09	0.27	0.14
		Saturate	GC	0.74	24.43	0.74	0.00	0.75	–26.88	1.71	–12.06	1.15	1.09	0.24	0.13
		Saturate	GC-MS (TIC)	0.93	57.46	0.74	0.00	0.62	–39.16	1.65	–15.44	1.14	1.09	0.30	0.17
		Saturate	GC-MS (*m*/*z* 85)	0.73	22.84	0.74	0.00	0.73	–28.60	1.37	–29.60	1.13	1.09	0.19	0.13
GH16	Shaximiao	Whole-oil	GC	0.42	0.00	/	/	1.40	0.00	1.69	0.00	1.09	1.07	0.14	0.09
		Saturate	GC	0.51	21.23	0.51	0.00	0.99	–29.39	1.44	–14.32	1.09	1.07	0.13	0.09
		Saturate	GC-MS (TIC)	0.65	52.49	0.45	–12.3	0.82	–41.41	1.48	–12.29	1.08	1.05	0.17	0.11
		Saturate	GC-MS (*m*/*z* 85)	0.51	20.70	0.52	1.40	0.96	–31.45	1.22	–27.48	1.07	1.06	0.11	0.09
GH18	Shaximiao	Whole-oil	GC	0.46	0.00	/	/	1.35	0.00	1.71	0.00	1.14	1.07	0.14	0.09
		Saturate	GC	0.48	3.35	0.48	0.00	1.07	–20.76	1.48	–13.28	1.07	1.06	0.13	0.09
		Saturate	GC-MS (TIC)	0.60	29.53	0.40	–15.58	0.90	–33.22	1.49	–12.80	1.10	1.07	0.17	0.11
		Saturate	GC-MS (*m*/*z* 85)	0.47	2.62	0.48	1.27	1.05	–22.16	1.26	–26.10	1.08	1.06	0.11	0.09
G101	Lianggaoshan	Whole-oil	GC	0.50	0.00	/	/	1.20	0.00	1.54	0.00	1.08	1.07	0.14	0.10
		Saturate	GC	0.53	5.48	0.53	0.00	1.05	–12.31	1.37	–10.67	1.09	1.06	0.14	0.10
		Saturate	GC-MS (TIC)	0.66	31.77	0.47	–11.80	0.88	–26.84	1.38	–10.42	1.10	1.05	0.17	0.13
		Saturate	GC-MS (*m*/*z* 85)	0.53	4.07	0.53	0.43	1.01	–15.31	1.13	–26.48	1.08	1.06	0.11	0.10
G13	Lianggaoshan	Whole-oil	GC	0.48	0.00	/	/	1.25	0.00	1.52	0.00	1.07	1.07	0.15	0.10
		Saturate	GC	0.62	29.01	0.62	0.00	0.85	–31.54	1.36	–10.70	1.08	1.06	0.13	0.09
		Saturate	GC-MS (TIC)	0.76	58.86	0.57	–8.50	0.74	–40.99	1.35	–11.67	1.10	1.07	0.18	0.13
		Saturate	GC-MS (*m*/*z* 85)	0.59	23.55	0.60	–2.70	0.86	–31.11	1.11	–27.31	1.08	1.05	0.11	0.10
G108X	Da’anzhai	Whole-oil	GC	0.49	0.00	/	/	1.24	0.00	1.52	0.00	1.08	1.06	0.14	0.10
		Saturate	GC	0.57	16.35	0.57	0.00	0.93	–24.71	1.24	–18.17	1.09	1.05	0.13	0.10
		Saturate	GC-MS (TIC)	0.70	42.84	0.51	–11.49	0.79	–36.29	1.31	–13.61	1.09	1.06	0.16	0.12
		Saturate	GC-MS (*m*/*z* 85)	0.56	13.89	0.57	–0.46	0.92	–25.74	1.10	–27.61	1.08	1.06	0.10	0.09
G111	Da’anzhai	Whole-oil	GC	0.54	0.00	/	/	1.20	0.00	1.76	0.00	1.12	1.07	0.14	0.09
		Saturate	GC	0.59	9.04	0.59	0.00	0.93	–22.99	1.49	–14.98	1.08	1.07	0.13	0.09
		Saturate	GC-MS (TIC)	0.69	28.89	0.50	–15.13	0.83	–31.22	1.56	–11.19	1.09	1.07	0.16	0.10
		Saturate	GC-MS (*m*/*z* 85)	0.52	–3.85	0.53	–10.22	1.02	–15.69	1.31	–25.65	1.07	1.07	0.11	0.08
JN35	Da’anzhai	Whole-oil	GC	0.50	0.00	/	/	1.22	0.00	1.49	0.00	1.01	1.07	0.15	0.11
		Saturate	GC	0.62	23.59	0.62	0.00	0.84	–31.26	1.17	–21.46	1.09	1.06	0.13	0.11
		Saturate	GC-MS (TIC)	0.77	52.84	0.57	–7.80	0.71	–41.77	1.26	–15.10	1.09	1.07	0.17	0.14
		Saturate	GC-MS (*m*/*z* 85)	0.60	20.07	0.61	–1.33	0.84	–31.01	1.04	–30.28	1.08	1.06	0.11	0.10
J94	Da’anzhai	Whole-oil	GC	0.50	0.00	/	/	1.22	0.00	1.41	0.00	1.23	1.06	0.16	0.12
		Saturate	GC	0.54	8.07	0.54	0.00	0.96	–21.36	1.34	–4.93	1.08	1.05	0.15	0.11
		Saturate	GC-MS (TIC)	0.62	22.87	0.42	–22.26	0.88	–28.16	1.37	–2.87	1.07	1.06	0.19	0.14
		Saturate	GC-MS (*m*/*z* 85)	0.47	–6.44	0.48	–11.69	1.06	–13.67	1.11	–21.15	1.08	1.05	0.12	0.11

a Notes: TAR = (nC_27_ + nC_29_ + nC_31_)/(nC_15_ + nC_17_ + nC_19_); CPI= ((nC_25_ + nC_27_ + nC_29_ + nC_31_ + nC_33_)/(nC_24_ + nC_26_ + nC_28_ + nC_30_ + nC_32_) + (nC_25_ + nC_27_ + nC_29_ + nC_31_ + nC_33_)/(nC_26_ + nC_28_ + nC_30_ + nC_32_ + nC_34_))/2; OEP = (nC_21_ + 6nC_23_ + nC_25_)/(4nC_22_ + 4nC_24_). TAR′ represents the corrected TAR value; the deviation rate is the deviation rate of the parameter values obtained by other testing methods relative to the parameter values obtained by full oil and gas chromatography; deviation rate′ represents the deviation rate of the parameter values obtained by other testing methods relative to the parameter values obtained by saturated hydrocarbon chromatography.

The *n*C_21_^–^/*n*C_22_^+^ ratio is also commonly used to indicate the relative contributions of lower aquatic organisms versus higher plants to the organic matter source.^4^ Because the abundances of *n*-alkanes below *n*C_13_ and above *n*C_34_ are very low, and to minimize errors arising from data processing for a more accurate comparison of parameter differences among analytical methods, we calculated this ratio using the carbon number range from *n*C_13_ to *n*C_34_. The *n*C_21_^–^/*n*C_22_^+^ ratios calculated from whole-oil GC are the highest, whereas the ratios derived from saturated-hydrocarbon-based analyses are significantly lower. Among the latter, the *n*C_21_^–^/*n*C_22_^+^ ratios calculated from the TIC chromatogram of saturated hydrocarbon GC-MS are the lowest, while those from saturated hydrocarbon GC are comparable to those from the *m*/*z* 85 mass chromatogram (Figure 4b). Therefore, when applying the TAR and *n*C_21_^–^/*n*C_22_^+^ parameters in practice, values calculated from saturated hydrocarbon GC and from the *m*/*z* 85 mass chromatogram can generally be used interchangeably, but they should not be directly mixed with values obtained from whole-oil GC or from the TIC chromatogram of saturated hydrocarbon GC-MS.

Differences in sample type (saturated hydrocarbons vs whole oil), analytical instruments, and even operating modes of the same instrument may cause systematic differences in the parameters obtained by the four analytical methods described above. For both TAR and *n*C_21_^–^/*n*C_22_^+^, the parameters calculated from saturated hydrocarbon GC show poor correlations with those from whole-oil GC, with correlation coefficients of 0.63 and 0.48, respectively (Figure 5a, b). Thus, the difference in sample type has a particularly pronounced effect on these parameters, and this effect is not systematic; it cannot be easily removed by simple correction.

**Figure 5:**
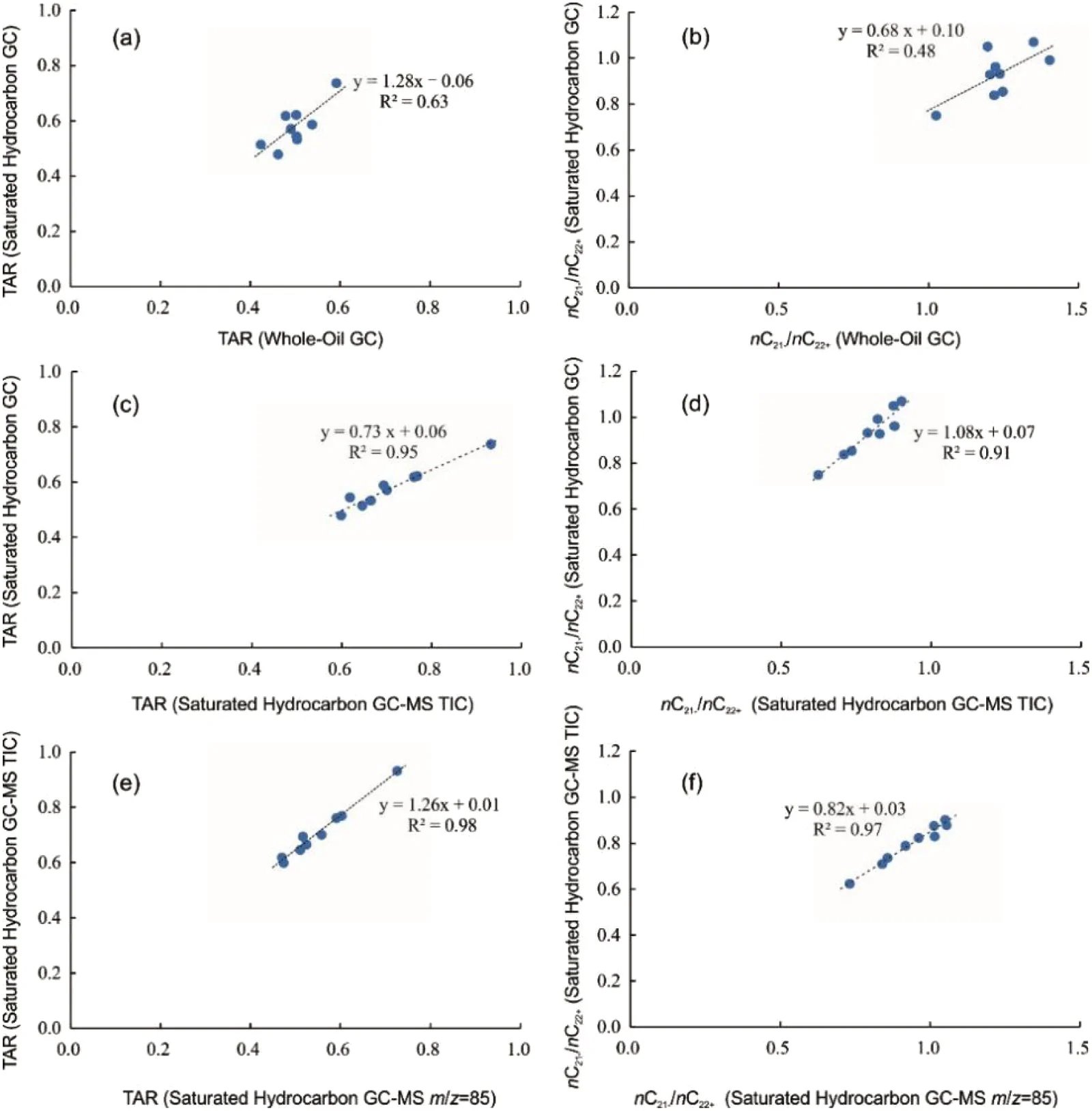
Cross plots of TARs (a, b, c) and *n*C_21_^–^/*n*C_22_^+^ ratios (d, e, f) obtained by different detection methods in Jurassic oils from the Sichuan Basin.

In contrast, parameters calculated from saturated hydrocarbon GC and from the TIC chromatogram of saturated hydrocarbon GC-MS show strong correlations (R^2^ > 0.90; Figure 5c, d), while those derived from the TIC and *m*/*z* 85 chromatograms exhibit even higher correlations (R^2^ > 0.95). Although systematic differences exist among the three approaches, these discrepancies can be substantially reduced by applying a simple calibration. For example, for sample G16, the TAR values obtained from saturated hydrocarbon GC and the TIC chromatogram are 0.74 and 0.93, respectively, corresponding to a correction factor of 0.19. After applying this correction to the TIC-derived TAR values, the magnitude of the average deviation from the saturated hydrocarbon GC results decreased from 22.67% to 13.10% (Table 2), demonstrating the effectiveness of the calibration. It should be noted that the correlations and correction factors fitted for geochemical parameters obtained by different experimental methods in this study are based on Jurassic crude-oil samples from the Sichuan Basin, and may not be applicable to samples from other regions. For analyses of samples from other areas, further fitting is required to obtain corresponding correction factors for parameters derived from different experimental methods. In addition, the correlation analyses presented in this study are based on nine crude oil samples. Therefore, these relationships mainly reflect the consistency among different analytical workflows for the investigated samples, and further validation using larger data sets is required to evaluate their broader applicability.

### 3.3 Pr/Ph Ratio

The Pr/Ph ratio is a commonly used parameter for indicating depositional environments.^1^ As shown in Figure 6a, the variation trends of Pr/Ph values obtained by different methods are consistent across samples. The Pr/Ph values derived from whole-oil GC are the highest, while those obtained from the *m*/*z* 85 mass chromatogram of saturated hydrocarbon GC-MS are the lowest. The Pr/Ph values from saturated hydrocarbon GC and from the TIC chromatogram of saturated hydrocarbon GC-MS are comparable to each other. Taking the Pr/Ph values from whole-oil GC as the reference standard, the Pr/Ph values from the *m*/*z* 85 mass chromatogram show high deviations, ranging from −30.3% to −21.2%. In contrast, the average deviation for Pr/Ph values obtained from saturated hydrocarbon GC and from the TIC chromatogram is −12.6%. Therefore, when using the Pr/Ph parameter in practice, values derived from saturated hydrocarbon GC and from the TIC chromatogram of saturated hydrocarbon GC-MS can generally be used interchangeably, but they should not be directly mixed with values from whole-oil GC or from the *m*/*z* 85 mass chromatogram. It is worth noting that Pr/Ph values obtained from the different analytical methods show excellent positive correlations, with correlation coefficients above 0.80 in all cases. Thus, through calibration, the systematic errors among these methods can also be substantially reduced, allowing parameters obtained by different methods to be used interchangeably.

**Figure 6:**
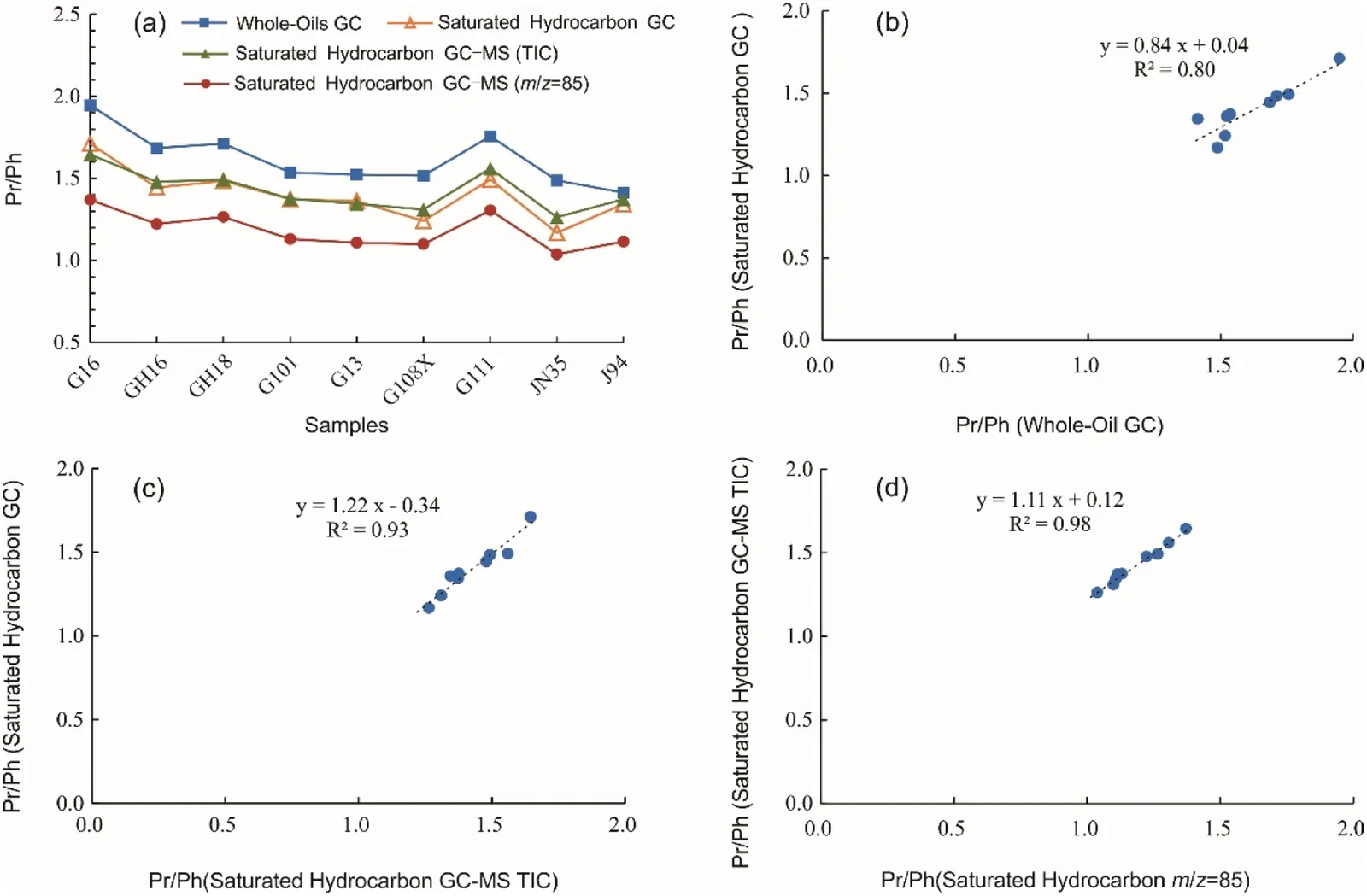
Variation of Pr/Ph ratios and comparison of detection methods in Jurassic oils from the Sichuan Basin. (a) Line chart showing the variation of Pr/Ph obtained by different detection methods across samples. (b) Cross-plot comparing Pr/Ph values from Whole-Oils GC and Saturated Hydrocarbon GC. (c) Cross-plot comparing Pr/Ph values from Saturated Hydrocarbon GC and Saturated Hydrocarbon GC-MS (TIC). (d) Cross-plot comparing Pr/Ph values from Saturated Hydrocarbon GC-MS (TIC) and Saturated Hydrocarbon GC-MS (*m*/*z* = 85).

### 3.4 CPI and OEP

The CPI (Carbon Preference Index) and OEP (Odd–Even Predominance) are commonly used for preliminary assessment of crude oil maturity.^18,19^ CPI or OEP values significantly greater than 1 or significantly less than 1 indicate low maturity.^5^ The calculation of CPI and OEP involves *n*-alkanes with carbon numbers above *n*C_21_, whereas the group-type fractionation discussed above mainly affects the *n*-alkane fraction below *n*C_16_. As shown in Figure 7, the CPI and OEP values for the Sichuan Basin crude oils are all around 1.1, and the differences among values obtained by different analytical methods are very small. Therefore, the choice of analytical method does not significantly affect these parameters, and the values derived from different methods can be used interchangeably in research.

**Figure 7:**
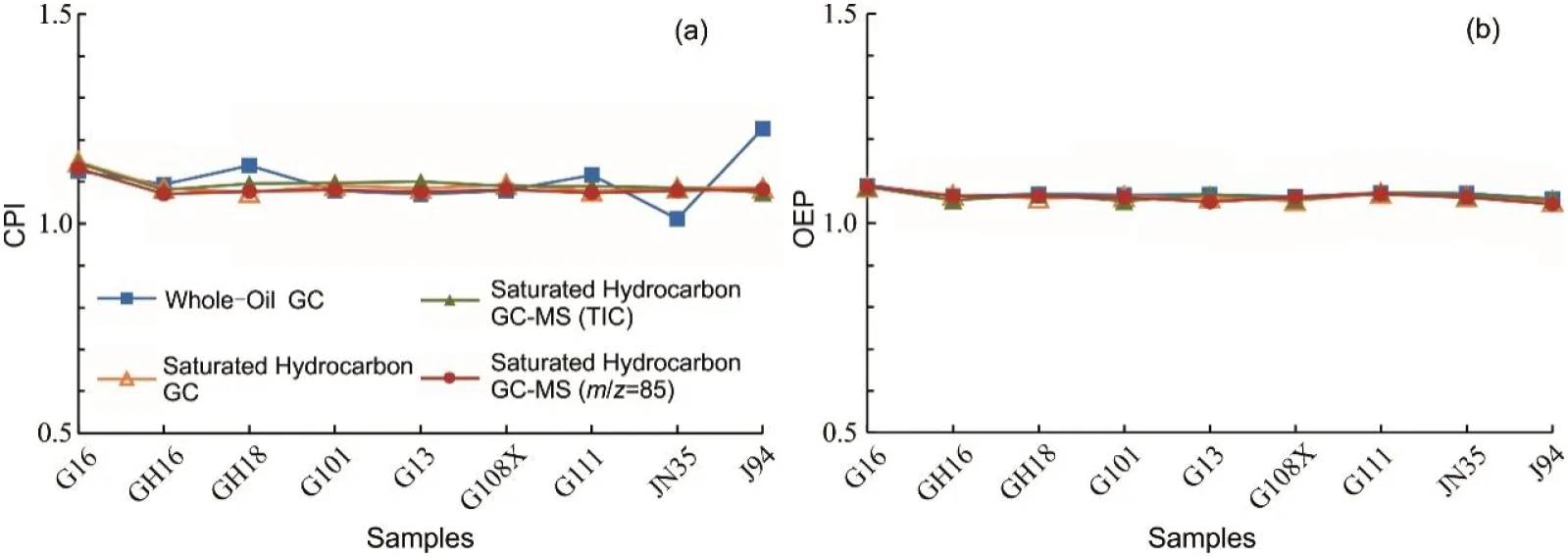
Line charts show the variation of CPI (a) and OEP (b) in Jurassic oils from the Sichuan Basin.

### 3.5 Pr/*n*C_17_ and Ph/*n*C_18_ Ratios

The Pr/*n*C_17_ and Ph/*n*C_18_ ratios can be used for oil family classification and source analysis, and cross-plots of these parameters are widely applied in studies of organic matter source, depositional environment, and biodegradation.^28–30^ As shown in Figure 8a, Pr/*n*C_17_ values derived from the TIC chromatogram of saturated hydrocarbon GC-MS are the highest, while those from the *m*/*z* 85 mass chromatogram are the lowest; however, the difference between the highest and lowest values is small, generally less than 0.1 (Figure 8a). The Ph/*n*C_18_ values obtained by different analytical methods also show very small differences (Figure 8b). As illustrated in Figure 9, the data points from different methods plot in a similar region, all indicating a mixed organic matter source for the crude oils. Therefore, the choice of analytical method does not significantly affect the Pr/*n*C_17_ and Ph/*n*C_18_ ratios either, and the values obtained by different methods can be used interchangeably in research without leading to erroneous conclusions.

**Figure 8:**
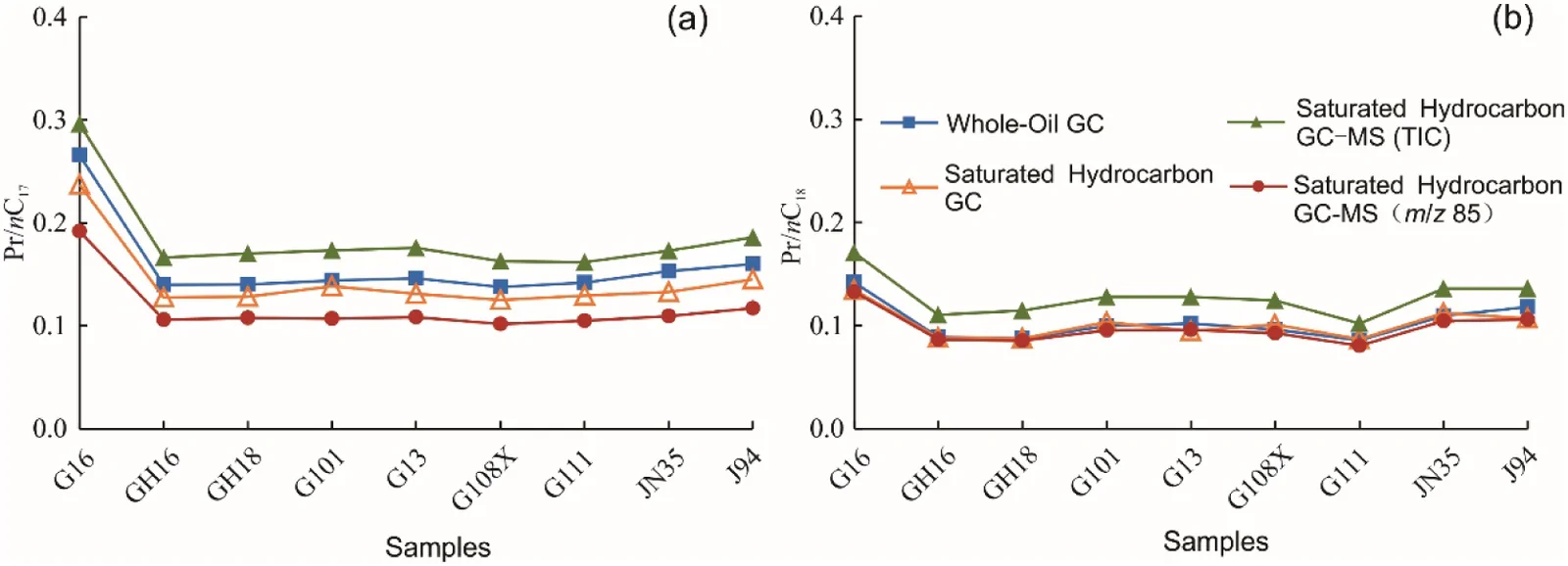
Line charts show the variation of Pr/*n*C_17_ (a) and Ph/*n*C_18_ (b) in Jurassic oils from the Sichuan Basin.

**Figure 9:**
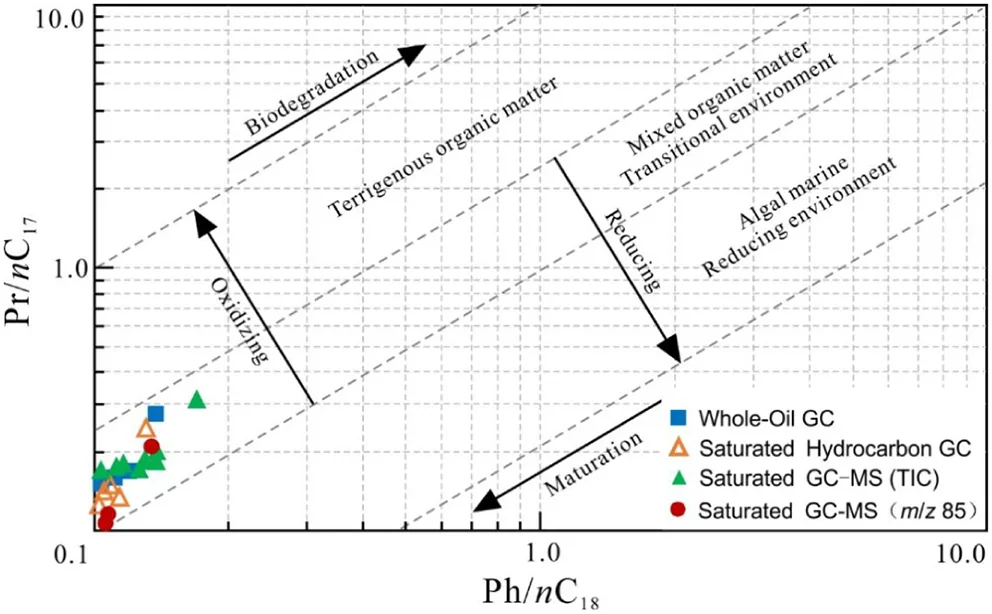
Cross plot of Pr/*n*C_17_ vs Ph/*n*C_18_ of Jurassic oils from the Sichuan Basin.

## 4 Conclusions

1 Whole-oil GC provides the most complete *n*-alkane distributions for the Jurassic crude oils from the Sichuan Basin, with *n*-alkanes mainly in the range of *n*C_3_ to *n*C_35_. In contrast, saturated hydrocarbon GC, the TIC chromatogram of saturated hydrocarbon GC-MS, and the *m*/*z* 85 mass chromatogram all only identify *n*-alkanes in the *n*C_14_ to *n*C_35_ range, and fail to detect low-molecular-weight hydrocarbons from *n*C_3_ to *n*C_13_. Group-type fractionation significantly alters *n*-alkane distribution patterns, leading to a marked reduction in the abundances of *n*C_14_–*n*C_16_ *n*-alkanes, and lowering the abundances of *n*C_3_–*n*C_13_ hydrocarbons below the detection limit.
2 For the calculation of TAR and *n*C_21_^–^/*n*C_22_^+^ parameters, values obtained from saturated hydrocarbon GC and from the *m*/*z* 85 mass chromatogram are comparable to each other, but differ notably from those derived from whole-oil GC and from the TIC chromatogram of saturated hydrocarbon GC-MS. The difference between whole-oil and saturated hydrocarbon sample types has a significant, nonsystematic (random) effect on these parameters, which is difficult to correct by simple calibration. However, differences arising from different analytical instruments or different operating modes of the same instrument are systematic. Parameters derived from saturated hydrocarbon GC, the TIC chromatogram, and the *m*/*z* 85 mass chromatogram show systematic differences, but after calibration (i.e., subtracting a correction factor), the comparability among these methods can be improved after calibration.
3 For the calculation of Pr/Ph, values derived from saturated hydrocarbon GC and from the TIC chromatogram of saturated hydrocarbon GC-MS are comparable, whereas those from whole-oil GC and from the *m*/*z* 85 mass chromatogram differ substantially. Because Pr/Ph values from different analytical methods show excellent positive correlations, after calibration the Pr/Ph values obtained by these methods are essentially interchangeable.
4 For CPI, OEP, Pr/*n*C_17_, and Ph/*n*C_18_, the values obtained by different analytical methods show very small differences; the choice of analytical method does not significantly affect these parameters.
5 It should be noted that the present study is based on a limited number of Jurassic light-oil samples from the Sichuan Basin. Therefore, the parameter-specific comparability and method-dependent relationships identified here should be further validated using oils with different maturities, biodegradation levels, compositions, and geological settings before being generalized to other petroleum systems.
